# Long-Term Changes in the Pollution of Warta River Bottom Sediments with Heavy Metals, Poland—Case Study

**DOI:** 10.3390/ijerph20105869

**Published:** 2023-05-18

**Authors:** Michał Fiedler

**Affiliations:** Department of Soil Science, Land Reclamation and Geodesy, Faculty of Environmental Engineering and Mechanical Engineering, Poznań University of Life Sciences, Piątkowska 94, 60-649 Poznań, Poland; michal.fiedler@up.poznan.pl

**Keywords:** heavy metals, bottom sediments, river, pollution

## Abstract

Variability in the heavy metal concentrations in aquatic environments may be influenced by a number of factors that may occur naturally or due to anthropopressure. This article presents the risk of contaminating Warta River bottom sediments with heavy metals such as As, Cd, Co, Cr, Cu, Hg, Mn, Ni, Pb, and Zn. Samples collected from 35 sites located along the river course were analysed over the period of 2010–2021. The calculated pollution indices are characterised by significant spatial variability that was additionally subject to changes in subsequent years. The analysis may have also been influenced by individual measurement results that, in extreme cases, may strongly deviate from the concentration values determined in the same site during the remaining years. The highest median concentrations of Cd, Cr, Cu, Hg, and Pb were in samples collected from sites that are surrounded by areas of anthropogenic land use. Samples from adjacent sites to agricultural areas showed the highest median concentrations of Co, Mn, and Ni, and Zn for those adjacent to forest areas. The research results indicate that, when analysing the degree of the risk of contaminating river bottom sediments with heavy metals, it is necessary to take into account long-term variability in metal concentrations. Taking into consideration data from only one year may lead to inappropriate conclusions and hinder planning protective measures.

## 1. Introduction

Heavy metals can be a significant contributor to the pollution of aquatic environments on account of their durability, toxicity, and capacity for bioaccumulation [1,2,3]. Metals may enter an aquatic environment as a result of the natural weathering process of the bedrock and the very strong influence of anthropogenic factors [4,5,6] that, in addition to constituting an independent source of pollution, may enhance natural processes. Sources of metal pollution may also depend on seasonal variability in precipitation [7]. A particularly large impact on the increase in heavy metal concentrations in alluvial deposits is attributed to industrial activity [8,9,10], intensive agriculture [11,12], intensive urbanisation [13,14], and mining activities [15,16,17]. The remobilisation of metals from older, often heavily contaminated bottom sediments may also play an important role [18,19]. The state of bottom sediments may be a valuable indicator describing the impact of natural and anthropogenic factors on an aquatic environment [20,21].

The distribution of heavy-metal concentration in bottom sediments may be influenced by the presence of argillaceous materials, organic matter, carbonate minerals, and other factors [22]. Adsorption on particles suspended in water plays a key role in the transfer of heavy metals to sediments [23]. Rivers transport heavy metals partially in dissolved form and partially absorbed on suspended matter that, falling to the bottom, accumulates them in sediments. At the same time, the spatial distribution and downstream transport of heavy metals captured in riverbed sediments are influenced by hydrodynamic processes [24,25,26]. Many studies have indicated that the concentrations of heavy metals in bottom sediments are influenced by hydrological conditions that control the physicochemical conditions and redox state of the sediments, consequently shaping various forms of metals and their transport [27]. Bottom sediments can act as both a reservoir, capturing contaminants, and a source of secondary pollution [28,29]. Most often, metals are subject to rapid deposition in riverbed sediments, and display much higher concentrations in sediments than those in river water [30]. At the same time, attention should be paid to the impact of natural and artificial water reservoirs on the transport of heavy metals in rivers [27,31]. The hazard of heavy metals may be particularly grave for bottom sediments in both rivers and lakes [32].

Generally used indicators for assessing the threat to an aquatic environment by heavy metals, such as the geoaccumulation index (Igeo), enrichment factor (EF), or potential ecological risk (RI), are based on comparing the concentrations of a given element in bottom sediments to the geochemical background (GB) value [33,34,35]. Due to the natural, regional variability of background values, it is inappropriate to use a single, generalised GB value for large-area analyses [36]. Geochemical background values should then be individually determined for each site. 

Wu et al. [37] suggests that the ongoing climatic changes, causing an increase in tem-perature and intense rainfall, may increase the content of heavy metals in water and sediments due to the enhanced leaching effect, non-point source pollution, and the release of heavy metals from sediments to water environments. The temporal and spatial variability of the degree of contamination of water and bottom sediments with heavy metals is affected by the type of pollution source [38]. So far, research on the impact of heavy metals on the quality of bottom sediments in the lowland areas of Poland has focused mainly on analysing spatial variability in individual years [39,40,41] while not taking into account long-term variability as a factor that may additionally affect heavy metal (HM) content in riverbed sediments. 

Many studies indicate that heavy metals can pose a threat to human health [42,43,44]. Heavy metals can enter the human body through the digestive system, skin, or inhalation [45]. The toxic effects of heavy metals affect different body organs. Gastrointestinal and renal disorders, nervous system disorders, skin lesions, vascular damage, immune system dysfunction, birth defects, anaemia, and cancer are examples of the complications of heavy metal toxicity [46,47,48]. Such adverse impacts of HMs on the environment and human health indicate the need for the continuous monitoring of the level of pollutants, and the identification of ways of migration into the environment.

This research was conducted in order to determine the temporal and spatial variability in the heavy metal contamination of the bottom sediments of the Warta River. Samples collected from 35 locations over 800 km of the river were used for the analysis. Variation analyses were performed for the metals Cd, Co, Cr, Cu, Hg, Mn, Ni, Pb, and Zn, and the metalloid As. The objectives of this work were as follows: (a) the statistical analysis of heavy metal concentrations in river bottom sediments over a 12-year study period, (b) the analysis of the spatial and temporal variability in sediment pollution level, and (c) determining the impact of land use in adjacent areas to the sampling sites on the pollution of the bottom sediments in the Warta River.

## 2. Materials and Methods

### 2.1. Object Description 

**Figure 1:**
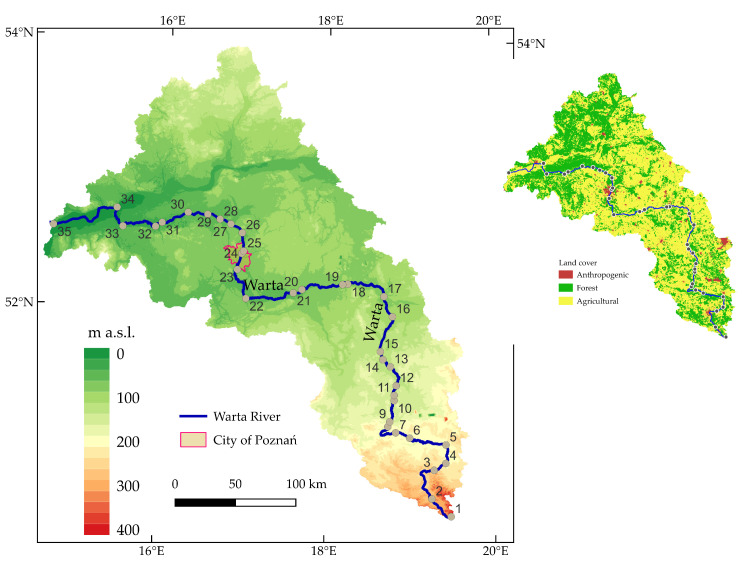
Map of the Warta River catchment and land cover of the analysed catchment according to the Corine land cover.

The Warta River is 808 km long and is the largest tributary of the Oder River. The riverhead is located at an altitude of about 380 m above sea level, and its discharge into the Oder River is at an altitude of 12 m above sea level (Figure 1). Its catchment area exceeds 54,500 km^2^. The largest city situated on the Warta River is Poznań that, together with its agglomeration, is home to almost 1 million residents (Places 23–25). 

In the soil cover of the Warta River basin, sandy soils constitute 45% of the total area, clay soils 41%, and organic and alluvial soils 14% [49]. According to the European Soil Database, the most common soils are Podzol—32%, Luvisols—30%, Fluvisols—12%, and Arenosols—12% [50]. The average annual air temperature for Poznań in the analysed period was 9.8 °C, and the average annual rainfall was 542 mm.

According to the Corine land cover (CLC) classification, agricultural soils dominate in the Warta River catchment area, accounting for 60.2% of the total area. Forest-covered and semi-natural areas account for 32.5%, areas of anthropogenic land use—5.6%, and surface waters—1.4% of the total area. In the rest of the area, wetlands and peatlands are found.

### 2.2. Materials

Data on the concentrations of As, Cd, Co, Cr, Cu, Hg, Mn, Ni, Pb, Zn and Fe in the bottom sediments of the Warta River were used in the analyses. The data obtained as part of the State Environmental Monitoring were made available by the Chief Inspectorate for Environmental Protection in Poland. Samples of bottom sediments were collected over 2010 and 2021 from a total of 35 sites (Figure 1). Sediment samples from each site were collected once a year from June to October. Table A1 summarises the years of sampling from individual sites. These sites were located mainly at the confluences of tributaries and downstream of cities with large industrial centres. For the chemical analyses, 4–5 samples of the top 5 cm layer of soil were collected from a 50 m section along the watercourse from each site. The samples from each site were mixed and sieved through a 2 mm mesh nylon sieve. Throughout the analysed period of 2010–2021, As, Cd, Co, Cr, Cu, Mn, Ni, Pb, Zn and Fe were determined using inductively coupled plasma atomic emission spectroscopy (ICP-OES) from obtained solutions after dissolving sediment samples with aqua regia. Hg in 2010–2012 and 2017 was determined from a solid sample via absorption spectrometry with concentration on an amalgamator. In 2013–2015 and 2018–2021, mercury was determined from a solid sample using atomic fluorescence spectrometry (ASF) with a DMA 80 mercury analyser.

Corine land cover (CLC 2018) data provided by the Chief Inspectorate for Environmental Protection were used to analyse the structure of land use in the areas adjacent to the measurement points. The CLC database contains vector land cover data updated for 2018 and allows for determining land use in five basic groups: anthropogenic land, agricultural land, forests and semi-natural ecosystems, wetlands, and water areas. This database provides a description of land cover and land use in Poland and the whole of Europe [51,52]. The geochemical background for each of the 35 sampling points was determined on the basis of the Polish Geochemical Atlas on a scale of 1:500,000.

### 2.3. Methodology

#### 2.3.1. Primary Statistics

The distribution of the concentrations of the heavy metals under study was determined using the Shapiro–Wilk test. The correlation values were determined using the Spearman rank method. The significance of the mean differences was calculated by means of the Wilcox test.

In order to isolate sampling sites displaying similar levels of heavy metal concentrations, cluster analysis was applied. The analysis was performed using Ward’s method. The number of clusters was determined using the silhouette and elbow method.

All general and statistical calculations, and the visualisation of the results, were performed using the R package version 4.2.2 (R Foundation for Statistical Computing, Vienna, Austria). 

#### 2.3.2. Geoaccumulation Index (Igeo)

The geoaccumulation Index (Igeo) was presented by Muller [33] and it allows for assessing the degree of pollution in relation to the value of the geochemical background (GB). The Igeo value is calculated from the following equation:

Igeo = log_2_(C_i_/1.5GB_i_) (1)

where C_i_ is the concentration of particular heavy metals (HMs) in the sediments, and GB_i_ is the value of the geochemical background for this metal. The geochemical background values (GB_i_) for each element were determined as a weighted average value for a 2 km long and 500 m wide buffer located above the sampling site according to the following formula:

*GB_i_ = Σ(GBC_i_·A_i_)/ΣA_i_* (2)

where GBC_i_ is the background content for each element, and A_i_ means the area per class in an analysed buffer [50]. Due to variability in the environmental parameters, GB values were determined for each sampling site. The average GB values were as follows: As–2.5 mg·kg^−1^, Cd–0.42 mg·kg^−1^, Co–1.49 mg·kg^−1^, Cr–4.74 mg·kg^−1^, Cu–5.49 mg·kg^−1^, Hg–0.046 mg·kg^−1^, Mn–194.5 mg·kg^−1^, Ni–3.53 mg·kg^−1^, Pb–16.83 mg·kg^−1^, Zn–40.49 mg·kg^−1^.

I_geo_ values allow for classifying the degree of pollution up to seven grades according to Table A2.

#### 2.3.3. Enrichment Factor (EF)

EF allows for defining anthropogenic causes of increased concentrations of heavy metals in sediments [53]. The EF is calculated by normalising the concentration values of the selected heavy metal to the concentrations of the reference element. These elements are characterised by very high stability in the soil that results from the lack of vertical mobility and/or degradation processes. The following are used as reference elements: aluminium (Al), iron (Fe), manganese (Mn), scandium (Sc), titanium (Ti), rubidium (Rb), and total organic carbon (TOC) and grain size [35]. In this work, iron (Fe) was adopted as the reference element.

The value of EF was calculated using the following formula:

EF = (C_i_/GB_i_)/(C_Fe_/GB_Fe_) (3)

where C_i_ is the concentration of the analysed heavy metal in the sediment sample, and GB_i_ is the geochemical background value for the sampling site. The values of C_Fe_ and GB_Fe_ denote the appropriate concentration of iron in the sample and the value of the geochemical background of Fe, respectively.

#### 2.3.4. Potential Ecological Risk (RI)

The potential ecological risk index (RI) was proposed by Håkanson [34,54] to assess the ecological risk resulting from concentrations of heavy metals in air, water, and soil. The value of the RI is calculated from the following formula:

(4) $$RI=\sum_{i=1}^{n}E_{r}^{i}$$

where $E_{r}^{i}$ is the environmental risk index calculated for each of *n* heavy metals obtained from the following equation:

(5) $$E_{r}^{i}=T_{r}^{i}\cdot {SP}_{i}$$

where: $T_{r}^{i}$ is the toxicity index of the i-th heavy metal, and SP_i_ is the value of the single pollution index (SPI). $T_{r}^{i}$ for As is 10, for Cd it is 30, for Co it is 5, for Cr it is 2, for Cu it is 5, for Hg it is 40, for Mn it is 1, for Ni it is 5, for Pb it is 5 and for Zn it is 1 [54]. The SP_i_ value is calculated from the following formula:

(6) $${SP}_{i}=C_{i}/R_{i}$$

where C_i_ is the concentration of the analysed heavy metal in the sediment sample and R_i_ is the reference value; here, the geochemical background value of each heavy metal GB_i_ was used. The limit values of the risk classes for individual metals $E_{r}^{i}$ and the overall RI risk are given in Table A1.

#### 2.3.5. Toxic Risk Index

The toxic risk index (TRI) allows for conducting a comprehensive assessment of the potential toxicity risk to aquatic organisms and was presented by MacDonald et al. [55]. TRI values were calculated on the basis of the threshold (TEL) and probable (PEL) values of heavy metal concentrations presented in Table 1. The TEL value corresponds to a concentration below which the effect of the metal is rarely observed. PEL, on the other hand, corresponds to the concentration above which side effects often occur [56]. The limit values for TRI risk classification are given in Table A1.

TRI values were calculated according to the following formula:

(7) $$TRI=\sum_{i=1}^{n}{TRI}_{i}$$

where TRI_i_ indicates the degree of heavy metal toxicity calculated from the following formula [57]:

(8) $${TRI}_{i}=\sqrt{\left({\left(C_{i}/TEL\right)}^{2}+{\left(C_{i}/PEL\right)}^{2}\right)/2}$$

#### 2.3.6. Land Use

The land use for each sampling point was determined for a buffer with a length of 2 km upstream of the watercourse from the sampling point and with a width of 500 m on the basis of CLC data and in accordance with the methodology presented by Fiedler [50]. The classification results are given in Table 2. Out of 35 sampling sites, 16 represent agricultural land, 8 are covered by forest, and 11 are of anthropogenic character.

## 3. Results

### 3.1. General Characteristics

Characteristic concentration values for the heavy metals under study, organic carbon (TOC), and pH in the collected riverbed sediment samples are presented in Table 3. The values for individual metals were characterised by high variability and fell within the following ranges: As, 0.198–22.9 mg·kg^−1^; Cd, 0.025–20.21 mg·kg^−1^; Co, 0.10–69.15 mg·kg^−1^; Cr, 0.782–33.16 mg·kg^−1^; Cu, 0.20–3170 mg·kg^−1^; Hg, 0.0005–1.57 mg·kg^−1^; Mn, 14.4–3644 mg·kg^−1^; Ni, 0.50–107.1 mg·kg^−1^; Pb, 0.50–1050 mg·kg^−1^; Zn, 0.25–1050 mg·kg^−1^. These values are similar to the concentrations of heavy metals in the bottom sediments of the Oder River, of which the analysed Warta River is a tributary [41,57]. The average concentration of heavy metals for the 12-year period of testing can be ordered in the following way: Mn > Zn > Cu > Pb > Cr > Ni > Co > As > Cd > Hg. The values of median concentrations can be ordered in the following way: Mn, 204.8 mg·kg^−1^, Zn, 40.25 mg·kg^−1^, Cr, 7.78 mg·kg^−1^, Pb, 7.71 mg·kg^−1^, Cu, 6.16 mg·kg^−1^, Ni, 3.92 mg·kg^−1^, Co, 1.59 mg·kg^−1^, As, 1.50 mg·kg^−1^, Cd, 0.25 mg·kg^−1^, Hg, 0.019 mg·kg^−1^. This pattern is similar to the presented distribution for the study of bottom sediments covering the entire territory of Poland [39,40,58].

**Figure 2:**
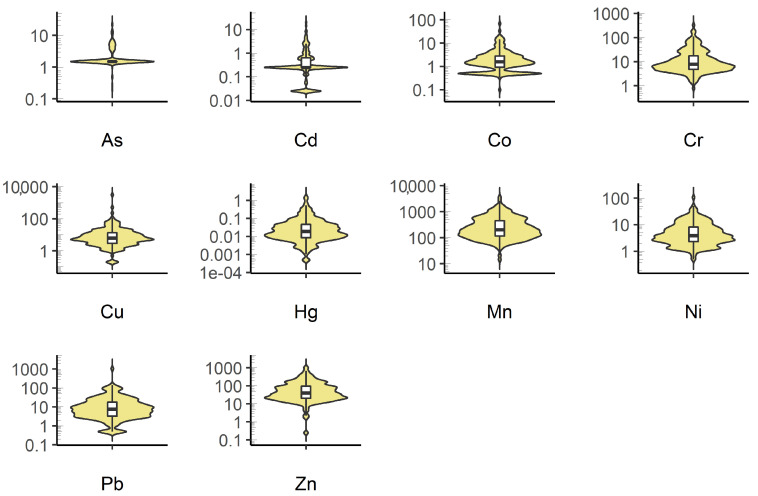
Variation in heavy metal concentrations (mg·kg^−1^) in the Warta river bottom sediments.

When analysing the average values of heavy metal concentrations in bottom sediments, it is noticeable that they are always higher than the average values of the geochemical background. This ratio is as follows: As–1.04, Pb–1.23, Hg–1.34, Mn–1.85, Cd–2.03, Ni–2.04, Co–2.05, Zn–2.19, Cr–4.03, Cu–5.32. At the same time, the values of the geochemical background for the analysed territory were lower than the values reported in the literature for European areas [59]. However, when comparing the median values of metal concentrations in sediments to the geochemical background, a different relationship can be discerned. In the case of As, Cd, Hg, and Pb, the median metal concentration constitutes about half of the background value; in the case of Zn, Co, Cu, Mn and Ni, the median heavy metal concentration is close to or slightly higher than the background value. Only in the case of Cr was the median concentration in sediments is 60% higher than the average median concentration of the geochemical background. The elevated mean values were influenced by single, very high concentrations of some metals in the collected bottom sediment samples, as shown in Figure 2. They occurred in different years and are associated with different sampling sites. This confirms the importance of point sources of pollution in the qualitative analyses of bottom sediments as emphasised by other authors [60,61,62]. The point locations of the sources may concern not only the spatial distribution, but also the temporal dimension. The analysis of the concentrations of the investigated metals indicates a similar distribution to the log-normal one (Figure 2). 

Correlation analysis using Spearman’s test showed a very strong correlation between almost all analysed heavy metals, at the level of *p* < 0.0001 (Table 4). Only the correlation between Hg and Cd concentrations was lower at the level of *p* < 0.05. HM concentrations, with the exception of cadmium and mercury, were also very strongly correlated with the total organic carbon (TOC). The correlation between the pH of the collected samples and HM concentrations, on the other hand, was only significant in the case of Co (*p* < 0.001).

### 3.2. Assessment of the Pollution of Bottom Sediments with Metals

**Figure 3:**
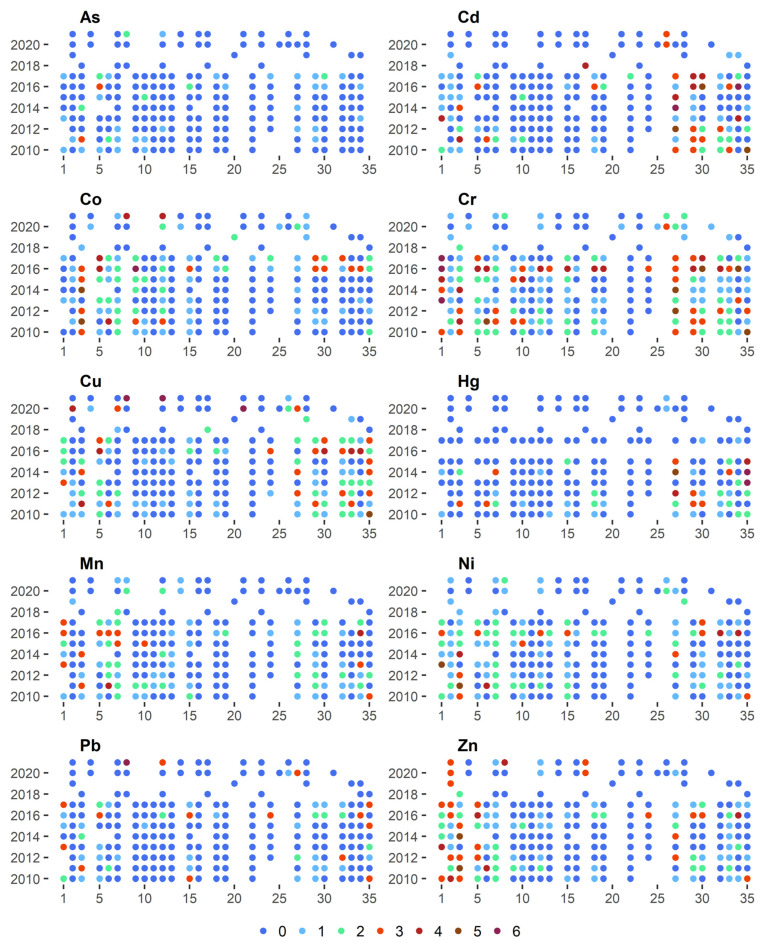
Classes of geoacumulation Index (I_geo_) for the 35 bottom sediment sampling sites for 2010–2021.

The geoaccumulation index (l_geo_) shown in Figure 3 could be used to assess the degree of the pollution of riverbed sediments with heavy metals [3]. The risk posed by individual metals exhibited fairly high temporal and spatial variability. The lowest risk was from As, whose concentration in the sediments was Class 3 for only two samples. On the other hand, as much as 82% of the samples with As concentrations were in Class 0. Low levels of pollution were also visible in the case of Pb, whose 78% concentration in the samples did not exhibit elevated values. Cr had the largest share in the pollution of bottom sediments, and only 33% of its samples are in Class 0. As much as 21% of its samples were classified as at least pollution Class 3.

Analysing the spatial variability of the l_geo_ index shows that the sites located along the middle course of the Warta River were characterised by the lowest level of pollution with all the metals. Concentrations of Cr, Co, and Cu in both the upper and lower sections of the river course, however, displayed elevated values. In the case of Cd, high l_geo_ values occur in sites located in the lower parts of the catchment (Sites 27, 29, 30). Hg concentrations in bottom sediments exhibited similar variability. However, in the case of Zn, high l_geo_ values could be observed mainly in the upper section of the watercourse (Sites 1–5).

Changes in metal concentrations in bottom sediments are also subject to time variability. The highest concentrations of virtually all metals occurred in 2016. This is especially evident in the case of Cr, where almost 60% of the samples collected were in at least class 3 of l_geo_. One can also notice sites where high l_geo_ values are maintained in consecutive years of observation. Assuming the occurrence of at least class 3 of l_geo_ in 4 of the 12 years of observation to be a threshold, the following sites can be distinguished in the case of Cd–27 and 29, Co–3, Cr–1, 3, 27 and 29, Cu–35, Ni–3, Zn–2, 3 and 5. This indicates the presence of a permanent source of pollution with individual metals in the vicinity of the sampling site.

**Figure 4:**
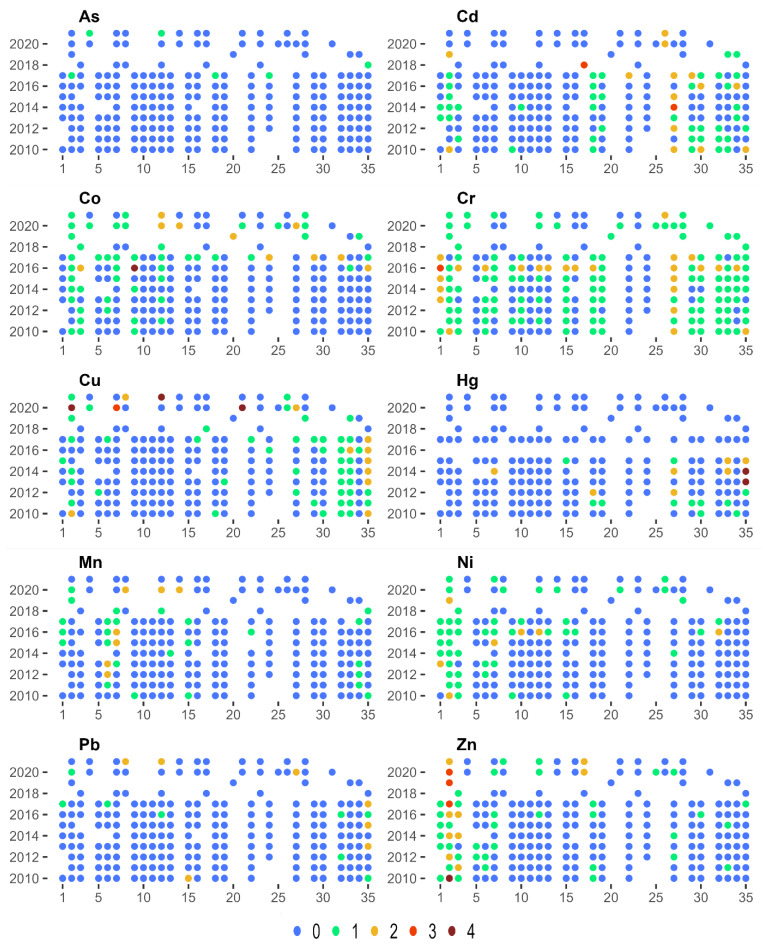
Enrichment Factor (EF) classes for 35 bottom sediment sampling sites for 2010–2021.

The calculated values of the enrichment index EF, similarly to I_geo_, showed spatial and temporal variability, depending on the analysed metal (Figure 4). In the case of Co, Mn, Ni, Zn, increased sediment pollution can be observed in the initial course of the river. This may have been due to the fact that these areas are highly industrialised, including ironworks and glassworks. On the other hand, in the case of Hg, the highest levels of pollution occur most often in the lower course of the river. Cr exhibited the highest levels of pollution in the entire analysed period and for all the analysed sites. At the same time, an increase in the EF value in the case of Co, Cr, and Ni could be observed over 2016–2017. 

### 3.3. Potential Environmental Risk of Heavy Metals

**Figure 5:**
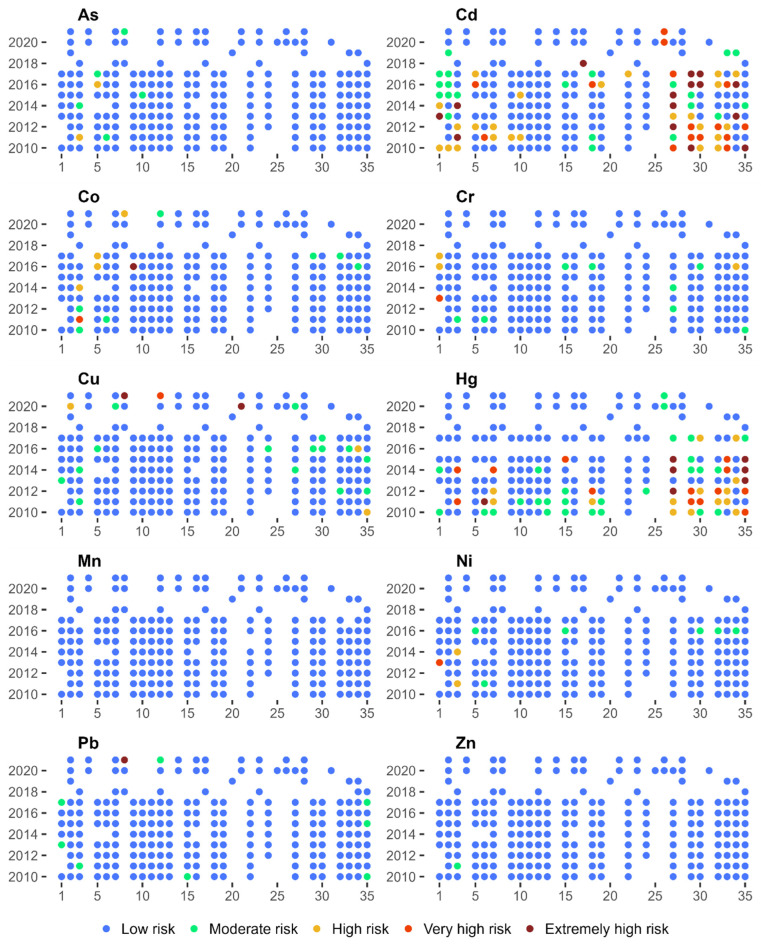
Ecological risk ($E_{r}^{i}$) classes for 35 bottom sediment sampling sites for 2010–2021.

**Figure 6:**
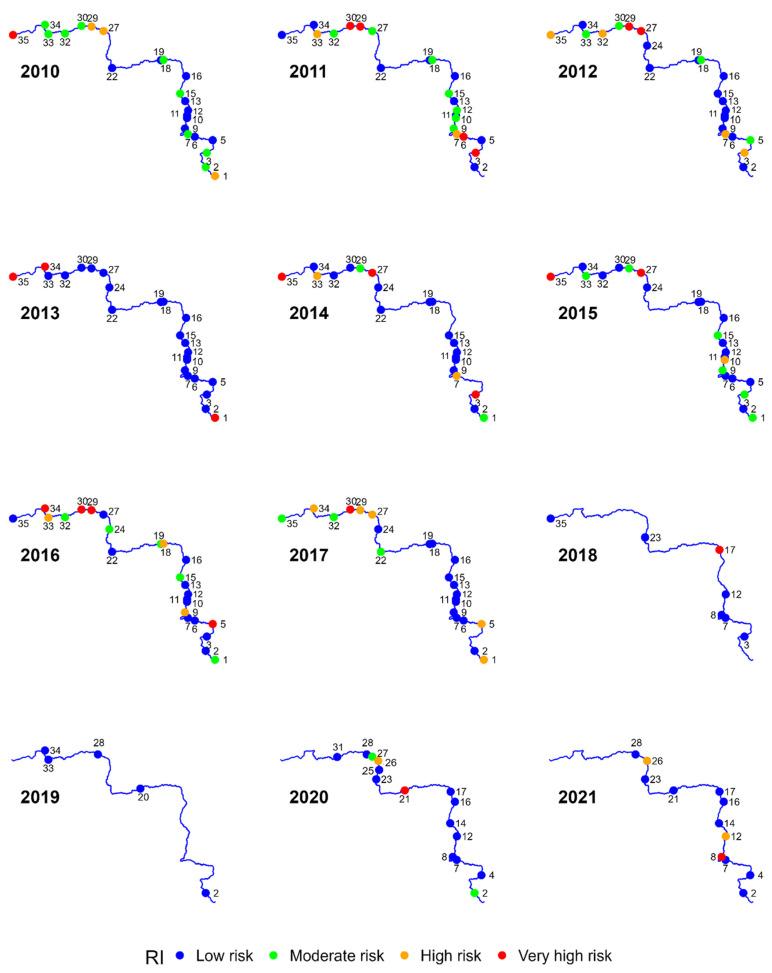
Ecological risk (RI) classes for 35 bottom sediment sampling sites for 2010–2021.

Another indicator that could be used to assess the degree of pollution in the bottom sediments of the Warta River is potential environmental risk indices $E_{r}^{i}$ and RI. The values of the $E_{r}^{i}$ index were measured for individual metals (Figure 5), while the *RI* (Figure 6) synthetically describes the risk posed by all metals. Since $E_{r}^{i}$ and *RI* take into account the degree of toxicity of individual metals, a different risk pattern could be observed than that in previously analysed indices. 

The highest ecological risk for the quality of bottom sediments is posed by the concentrations of Cd and Hg, especially for sites located in the lower section of the Warta River (Figure 5). Adopting, as in the case of *I_geo_*, the occurrence of at least high environmental risk lasting 4 years as the threshold ($E_{r}^{i}$ > 80), Sites 3, 27, 29, 30, 32, 33 for Cd, and Sites 27, 33, 35 for Hg are under the greatest threat. Areas adjacent to Sites 3, 27, and 33 are subject to anthropogenic land use, while those adjacent to Sites 29, 30, and 35 are used for agricultural purposes. In the case of the other metals, they do not present a significant environmental risk. The high risk of pollution of bottom sediments with Cd and Hg stems from their high toxicity, which is also confirmed by the research of Cui et al. (2019) [63]. 

The analysis of the value of synthetic index RI allows for pinpointing the sites that display the characteristics of ecological hazard posed by heavy metals (Figure 6). These sites are located mainly along the lower course of the river, downstream from Site 27. In general, heavy metal concentrations in 61% of the samples collected can be classified as low risk (RI < 150). 17% of the samples indicate average ecological hazard (150 < RI < 300), and a total of 22% of the samples were characterised by high and very high hazards.

Analysing the course of changes in the RI in the upper course of the Warta River, in the following years, a certain trend can be observed indicating the movement of pollutants along the course of the river. In 2013, Site 1 was very high risk. A similar level of risk was observed in 2014 in Site 3, and in 2016 in Site 5. In the following year, the level of risk in Site 5 decreased to a high level. The association of the cases with one source of contamination may be indicated by the fact that in each of the analysed sites, the metal that increased the risk of RI was cadmium. This process may suggest the influence of factors related to the transport of sediments with flowing water. However, the rate of pollutant movement related to the speed of water flow results from many factors related to the morphometry of the terrain, the state of the river or meteorological conditions [37,64]. However, in the lower course of the river, this phenomenon is difficult to observe. This may be due to the predominance of deposition processes associated with lower water flow velocity in this section of the river. Jaskuła and Sojka [40] point to a similar pattern for the Odra River and explain it by the presence of point and area sources of pollution along the river. 

### 3.4. Influence of Heavy Metals on Aquatic Organisms

**Figure 7:**
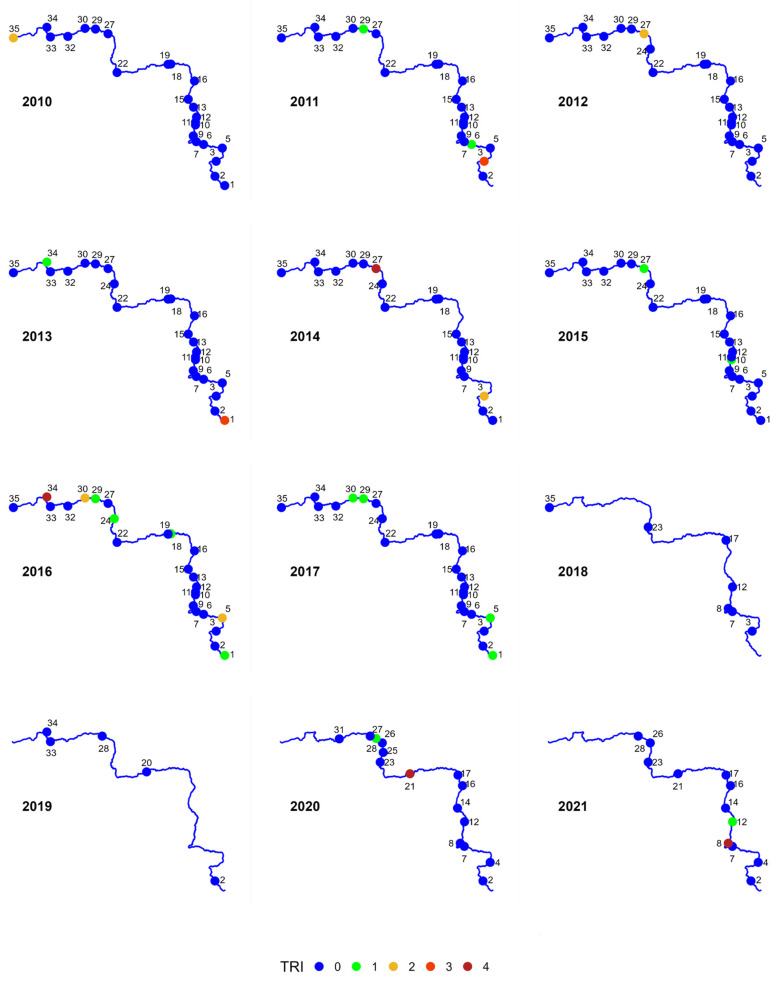
Toxic risk index (TRI) classes for 35 bottom sediment sampling sites for 2010–2021.

The toxicity risk of heavy metals contained in bottom sediments can be assessed using the Toxic Risk Index (TRI; Figure 7). The calculations of the index take into account the reference of individual concentration values to the concentrations corresponding to TEL and PEL. Of the 224 samples collected during the 12—year study period, 88% of them do not display any risk of toxicity (class 0), and another 7% carry a low risk of toxicity (class 1). Only 4 of the samples collected show a very high risk (TRI > 20, class 5). The highest TRI value of 73.3 was noted for bottom sediments collected in 2021 in Profile 8. Such high value of the index was caused by a very high concentration of copper in the collected sample. The toxicity index for this metal reached TRI_Cu_ = 75.2. In the remaining years, the TRI for this site did not indicate any risk of toxicity (class 0). The remaining three samples also sporadically indicated a very high risk of toxicity occurring. In 2020, the TRI value in a sample collected from Site 21 reached 41.4. The metal that influenced the high value of the index was lead. In 2016, a sample taken from Site 35 and in 2014 a sample taken from Site 27 were qualified for Class 5. In both cases, cadmium was responsible for the high value of the TRI.

### 3.5. Analysis of the Impact of Land Use on Heavy Metal Concentrations

The analysis of the impact of the areas adjacent to the site of collecting bottom sediment samples was carried out by classifying the use of a buffer with a length of 2 km and a width of 500 m, located upstream from the sampling site [50]. This allowed for isolating three types of land use: agricultural areas, forest areas and areas of anthropogenic land use (Table 2). 

**Figure 8:**
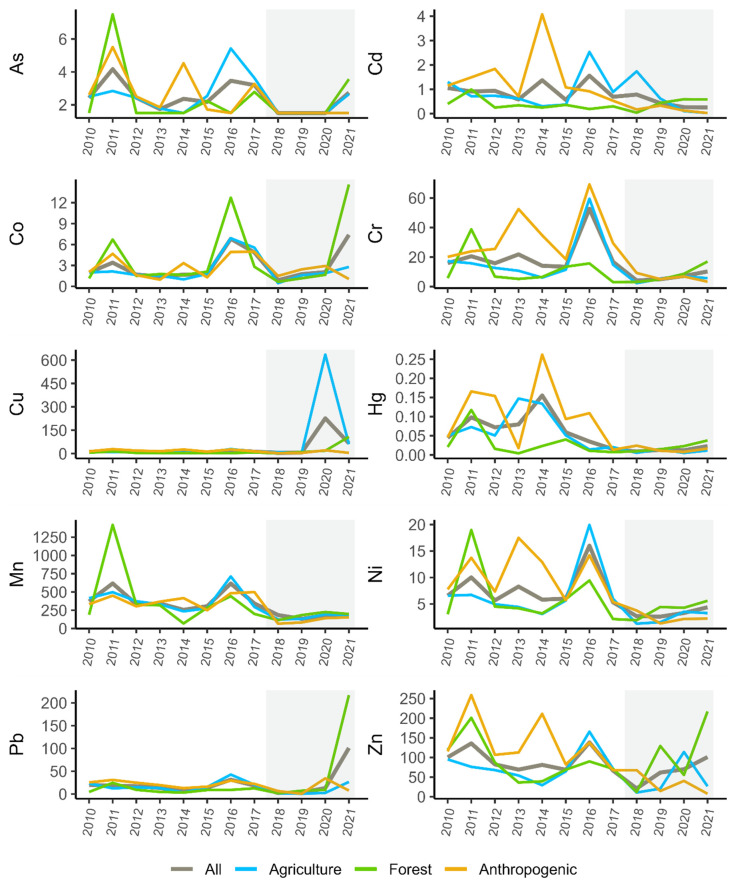
Average concentrations of heavy metals (mg·kg^−1^) for various types of land use adjacent to the sampling sites for 2010–2021.

**Figure 9:**
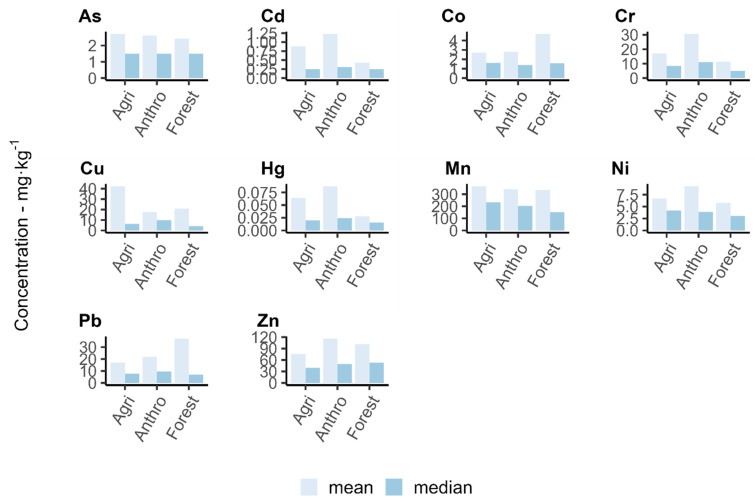
Mean and median concentrations (mg·kg^−1^) of heavy metals in the bottom sediments of the Warta River for different types of land use adjacent to the sampling sites.

The average annual concentrations of heavy metals in bottom sediments for different forms of land use in the areas adjacent to the sampling site presented in Figure 8 are characterised by fairly high variability. In the case of agricultural areas, the highest mean values for the period of metal concentration research referred to As, Cu, and Mn. In the case of anthropogenic land use—Cd, Cr, Hg, Ni, and Zn, and in the case of forestry—Co and Pb (Figure 9). However, in no case were the differences between the mean values for different forms of land use statistically significant. The average concentrations, of, e.g., Co, Cu or Pb, were very strongly influenced by the single very high quantification values of these metals in the samples. On the other hand, when analysing the median values, it can be noted that for sites adjacent to agricultural areas, Co, Mn, and Ni reached the highest concentration values, and in the case of forest environment it was Zn. In the case of anthropogenic environments, these were Cd, Cr, Cu, Hg, and Pb. 

**Figure 10:**
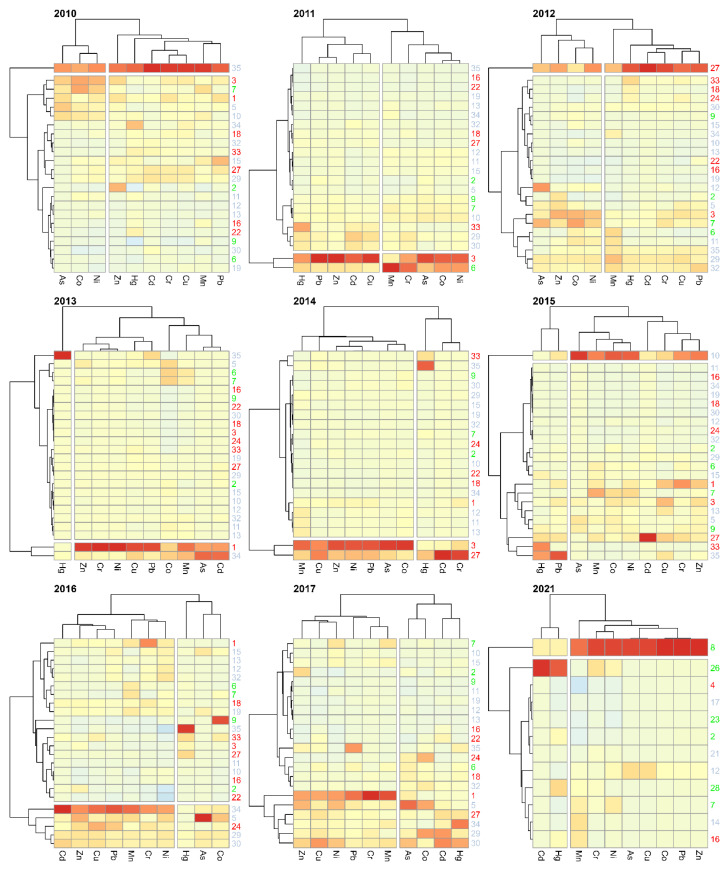
Heatmap of clustering heavy metals concentrations.

Figure 10 presents heat maps and clustering of sampling points and metal concentrations for 2010–2017 and 2021. Due to the insufficient sample size, such classification for 2018–2020 was not possible. In the figure, grey is used to mark the sampling points associated with agricultural use, green—forestry, and red the anthropogenic land use. Due to the insufficient sample size, the analysis was not possible for 2018–2020. The groupings allowed for establishing in each year a maximum of two groups of sampling sites characterised by a similar distribution of heavy metal concentrations. Most often, one of the groups was formed by one or two sampling sites. The lack of repeatability of the sites isolated in this way over the consecutive years should be noted. There was also no connection with the land use of adjacent areas. The following sites were isolated as statistically different from the remaining ones: in 2010–site 35; in 2011–sites 3 and 6; in 2012–27; in 2013–1 and 34; in 2014–3 and 27; in 2015–10; in 2016–5, 24, 29, 30, and 34; in 2017, no sites were isolated; in 2021–8. Similar conclusions, indicating the lack of relationship between the use of catchment areas and the concentrations of heavy metals in river bottom sediments of Polish rivers, were obtained by Sojka and Jaskuła [39]. The data, covering a period of one year, used by them for the analysis, covered rivers located throughout Poland and partly in neighboring countries. At the same time, it may suggest that the sources of contamination of bottom sediments by HM are mainly point sources. This is also confirmed by the results of research on contamination of river sediments obtained by Jaskuła and Sojka [40], who additionally indicate fluvial processes as a modifying factor. The results of earlier studies also indicated that the concentrations of heavy metals are mainly influenced by point sources [65,66].

**Figure 11:**
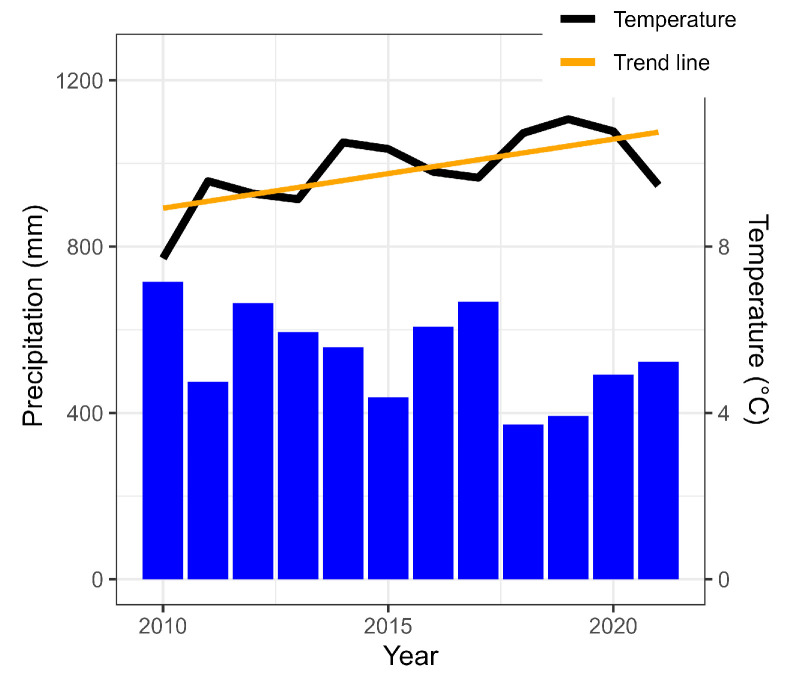
Annual precipitation sums and average annual air temperatures for Poznań in 2010–2021.

Climate change is also mentioned as a factor affecting the level of bottom sediment pollution [37]. Figure 11 presents average annual air temperatures and annual precipitation totals for Poznań. In the analysed period, there was a clear upward trend in air temperatures, while precipitation showed the opposite trend. This could indicate a reduction in the amount of water forming surface runoff, which would also reduce the supply of pollutants delivered with them to surface waters. However, the observed changes in meteorological conditions indicate an increasing number of extreme precipitation events, even with decreasing annual rainfall [67,68]. This may result in an increase in surface runoff and erosion processes [69], and consequently in an increased supply of pollutants to surface waters [70]. As a result, there may be an increased risk of pollution, especially in waters adjacent to urbanised areas [71]. On the other hand, higher temperatures in winter and the lack of snow cover mean that, in these periods, surface runoff also occurs more often. This causes increased leaching of pollutants from agriculturally used areas that are not protected by vegetation in these periods [72].

## 4. Conclusions

The results of the 12–year study indicate high spatial and temporal variability of heavy metal concentrations in the bottom sediments of the Warta River. The analysis of clusters does not indicate the existence of relationships between the concentrations of heavy metals in bottom sediments and the land use of the adjacent areas. However, some impact of the land use in the adjacent areas on the average concentrations of metals in the analysed period was found, but the differences between the mean HM concentrations for each form of land use were not statistically significant. For the areas of anthropogenic land use, Cd, Cr, Cu, Hg, and Pb reached the highest median concentrations, for agricultural areas–Co, Mn, and Ni, and for forest areas–Zn. In the case of As, the median concentration was equal for all forms of land use in the adjacent areas. 

The calculated indices of I_geo_, EF, $E_{r}^{i}$, Ri, and TRI in most of the samples collected indicate no or slight pollution of the sediment with metals. Single, very high values of the analysed indices occur in different years and in sites where the land use in the adjacent area takes various forms. These points require detailed analyses of the origin of the contaminants that is only possible immediately after pollution is detected. 

The conducted analysis indicates that in the upper part of the river course, the influence of fluvial processes on the movement of pollutants can be observed, while in the lower course, deposition processes play a greater role. 

The results of the analyses indicate that the use of data from different periods for the analyses may lead to different conclusions on the degree of risk for bottom sediments pollution with heavy metals.

## Figures and Tables

**Table 1:** Threshold (TEL) and probable effect (PEL) concentrations of heavy metals (mg·kg^−1^).

Heavy Metal	TEL	PEL
As	7.2	41.6
Cd	0.68	4.2
Cr	52.3	160.4
Cu	18.7	108.2
Ni	15.9	42.8
Pb	30.2	112.2
Zn	124	271

**Table 2:** Classification of sampling sites due to the use of adjacent areas.

Area Use	Site No.
Agricultural	5, 10, 11, 12, 13, 14, 15 17, 19, 21, 29, 30, 31, 32, 34, 35
Forest	2, 6, 7, 8, 9, 23, 26, 28
Anthropogenic	1, 3, 4, 16, 18, 20, 22, 24, 25, 27, 33

**Table 3:** Characteristic concentrations of heavy metals (mg·kg^−1^), TOC, and pH in the Warta River bottom sediments in 2010–2021, and geochemical background values (mg·kg^−1^) for soils in the areas adjacent to the sampling sites.

	As	Cd	Co	Cr	Cu	Hg	Mn	Ni	Pb	Zn	TOC	pH
	Sampling sites											
min	0.198	0.025	0.10	0.782	0.20	0.0005	14.4	0.50	0.50	0.25	0.05	6.10
mean	2.59	0.852	3.05	19.08	29.23	0.0615	359.9	7.20	20.75	88.59	1.86	7.66
median	1.5	0.25	1.59	7.78	6.16	0.0190	204.8	3.92	7.71	40.25	0.51	7.62
max	22.9	20.21	69.15	33.16	3170	1.57	3644	107.1	1050	1050	51.50	8.93
sd	2.87	2.01	5.90	33.9	214.6	0.1614	428.5	10.1	72.8	138.6	4.20	0.42
skew	4.05	6.13	7.39	5.22	14.05	6.53	3.54	5.41	12.61	4.09	8.06	−0.32
curtosis	20.6	47.1	72.1	36.6	202	50.3	18.6	44.1	174.1	21.4	86.7	1.2
IQR	0	0.389	1.92	12.7	10.7	0.0388	334	5.86	14.5	74.8	1.69	0.50
	Geochemical background											
mean	2.50	0.42	1.49	4.74	5.49	0.046	194.5	3.53	16.83	40.49		
sd	0	0.43	0.71	3.41	2.13	0.024	49.3	1.43	12.38	15.83		

**Table 4:** Spearman correlation results.

	As	Cd	Co	Cr	Cu	Hg	Mn	Ni	Pb	Zn	TOC
Cd	0.38										
Co	0.57	0.29									
Cr	0.56	0.63	0.59								
Cu	0.47	0.61	0.48	0.73							
Hg	0.37	0.53	0.16	0.64	0.57						
Mn	0.43	0.41	0.60	0.67	0.44	0.37					
Ni	0.57	0.47	0.76	0.82	0.59	0.48	0.78				
Pb	0.55	0.57	0.52	0.74	0.76	0.61	0.53	0.65			
Zn	0.59	0.57	0.58	0.73	0.68	0.53	0.56	0.74	0.76		
TOC	0.28	0.08	0.41	0.34	0.37	0.19	0.33	0.37	0.29	0.31	
pH	−0.11	0.01	−0.26	−0.05	−0.03	0.13	−0.02	−0.02	−0.13	−0.10	−0.11

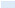
 *p* < 0.0001, 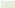 *p* < 0.001, 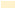 *p* < 0.01, 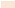 *p* < 0.05.

## Data Availability

The Corine Land Cover 2018 in Poland project was implemented by the Institute of Geodesy and Cartography, and financed by the European Union. The results of the project were obtained from the website of the Chief Inspectorate of Environmental Protection https://clc.gios.gov.pl (accessed on 8 May 2023) (in polish). Data on the pollution of bottom sediments are available on https://www.gov.pl/web/gios/monitoring-osadow-dennych (accessed on 8 May 2023) (in Polish).

## Appendix A

**Table A1:** Sampling sites of bottom sediments in 2010–2021.

Year	Sampling Sites
2010	1, 2, 3, 5, 6, 7, 9, 10, 11, 13, 15, 16, 18, 19, 22, 27, 29, 30, 32, 33, 34, 35
2011	2, 3, 5, 6, 7, 9, 10, 11, 13, 15, 16, 18, 19, 22, 27, 29, 30, 32, 33, 34, 35
2012	2, 3, 5, 6, 7, 9, 10, 11, 13, 15, 16, 18, 19, 22, 24, 27, 29, 30, 32, 33, 34, 35
2013	1, 2, 3, 5, 6, 7, 9, 10, 11, 13, 15, 16, 18, 19, 22, 24, 27, 29, 30, 32, 33, 34, 35
2014	1, 2, 3, 7, 9, 10, 11, 13, 15, 18, 19, 22, 27, 29, 30, 32, 33, 34, 35
2015	1, 2, 3, 5, 6, 7, 9, 10, 11, 13, 15, 16, 18, 19, 22, 24, 27, 29, 30, 32, 33, 34, 35
2016	1, 2, 3, 5, 6, 7, 9, 10, 11, 13, 15, 16, 18, 19, 22, 24, 27, 29, 30, 32, 33, 34, 35
2017	1, 2, 3, 5, 6, 7, 9, 10, 11, 13, 15, 16, 18, 19, 22, 24, 27, 29, 30, 32, 33, 34, 35
2018	3, 7, 8, 12, 17, 23, 35
2019	2, 20, 23, 33, 34
2020	2, 4, 7, 8, 12, 14, 16, 17, 21, 23, 25, 26, 27, 28, 31
2021	2, 4, 7, 8, 12, 14, 16, 17, 21, 23, 26, 28

**Table A2:** Classes of pollution indices.

Pollution Index	Class	Value	Description	Reference
I_geo_	0	I_geo_ ≤ 0	Practically unpolluted	Müller (1986) [73]
	1	0 < I_geo_ ≤ 1	Unpolluted to moderately polluted	
	2	1 < I_geo_ ≤ 2	Moderately polluted	
	3	2 < I_geo_ ≤ 3	Moderately severe polluted	
	4	3 < I_geo_ ≤ 4	Severely polluted	
	5	4 < I_geo_ ≤ 5	Very severely polluted	
	6	5 < I_geo_	Extremely polluted	
EF	0	EF ≤ 2	Deficiency to minimal enrichment	Barbieri (2016) [35]
	1	2 < EF ≤ 5	Moderate enrichment	
	2	5 < EF ≤ 20	Significant enrichment	
	3	20 < EF ≤ 40	Very high enrichment	
	4	40 < EF	Extremely high enrichment	
$E_{r}^{i}$	0	$E_{r}^{i}$ ≤ 40	Low risk	Håkanson (1980) [34]
	1	40 < $E_{r}^{i}$ ≤ 80	Moderate risk	
	2	80 < $E_{r}^{i}$ ≤ 160	High risk	
	3	160 < $E_{r}^{i}$ ≤ 320	Very high risk	
	4	320 < $E_{r}^{i}$	Extremely high risk	
RI	0	RI ≤ 150	Low risk	Håkanson (1980) [34]
	1	150 < RI ≤ 300	Moderate risk	
	2	300 < RI ≤ 600	High risk	
	3	600 < RI	Very high risk	
TRI	0	TRI ≤ 5	No toxicity risk	Zhang et al. (2016) [74]
	1	5 < TRI ≤ 10	Low risk	
	2	10 < TRI ≤ 15	Moderate risk	
	3	15 < TRI ≤ 20	Considerable risk	
	4	20 < TRI	Very high risk	
